# Mortality in children with cancer and SARS-CoV-2 in Latin America: A systematic review

**DOI:** 10.3389/fped.2022.928612

**Published:** 2022-08-09

**Authors:** Elisa Dorantes-Acosta, Diana Ávila-Montiel, Jesús Domínguez Rojas, Patricia Parra-Nigañez, Liliana Velasco-Hidalgo, Sergio Arias, Lourdes Gutiérrez-Rivera, Luis Juárez-Villegas, Horacio Márquez-González

**Affiliations:** ^1^Biobanco de Investigación en Células Leucémicas, Hospital Infantil de México Federico Gómez, México City, Mexico; ^2^Departamento de Onco-Hematología, Hospital Infantil de México Federico Gómez, México City, Mexico; ^3^Investigación Clínica, Hospital Infantil de México Federico Gómez, México City, Mexico; ^4^Unidad de Cuidados Intensivos Pediátricos, Hospital Nacional Edgardo Rebagliati Martins, Lima, Peru; ^5^Departamento de Oncología Pediátrica, Hospital del Niño “Dr. Ovidio Aliaga Uria”, La Paz, Bolivia; ^6^Departamento de Oncología Pediátrica del Instituto Nacional de Pediatría, Ciudad de Mexico, Mexico; ^7^Departamento Programas de Salud, INER Emilio Coni, Epidemiología y Salud Pública, Facultad de Bioquímica y Ciencias Biológicas (FBCB), Universidad Nacional del Litoral (UNL), Santa Fe, Argentina; ^8^Oncología Pediátrica, Centro Médico Nacional Siglo XXI, UMAE Hospital de Pediatría, Instituto Mexicano del Seguro Social (IMSS), México City, Mexico; ^9^Cardiopatías Congénitas, Centro Médico Nacional Siglo XXI, Hospital de Cardiología, Instituto Mexicano del Seguro Social (IMSS), México City, Mexico

**Keywords:** SARS-CoV-2, COVID-19, children, pediatric, cancer, Latin America

## Abstract

The new COVID-19 disease is caused by a novel coronavirus (SARS-CoV-2), that probably originated in Wuhan, China, and has currently infected 505,817,953 people and caused 6,213,876 deaths in the world. On the American continent, 152,265,980 cases and 2,717,108 deaths have been reported to WHO (World Health Organization). The Latin America and the Caribbean (LAC) region presents an epidemiological challenge due to its population's heterogeneity and socioeconomic inequality. A particularly vulnerable population is that of children with cancer, and their mortality from COVID-19 has been reported to be 3.6% globally. This work aimed to study the lethality of SARS-CoV-2 infection in children with cancer in the Latin American region. Our objective was to systematically review published scientific literature and search hospital databases in Latin America to explore mortality in this region. A median of mortality of 9.8% was found in the articles analyzed. In addition, we collected five databases from Latin American hospitals. We concluded that there was an underestimation in the mortality registry of this group of patients in the analyzed region. Therefore, although the causes are unknown, it is necessary to strengthen the case-reporting system to determine the reality in complex and particular areas such as Latin America.

## Introduction

The new COVID-19 disease is caused by a novel coronavirus (SARS-CoV-2) that possibly originated in Wuhan, China; in December 2019, the Wuhan health authorities detected a few cases of atypical pneumonia that was discovered to be caused by a novel coronavirus. It probably jumped from an animal reservoir to a human during the first week of November 2019 (1).

**Globally**, by **April 22, 2022**, there have been **505,817,953 confirmed cases** of COVID-19, including **6,213,876 deaths**, reported to WHO. On the American continent, 152,265,980 cases and 2,717,108 deaths have been reported. The Latin America and the Caribbean (LAC) region presents an epidemiological challenge due to the heterogeneity of its population and socioeconomic inequality. In LAC, the first confirmed case of COVID-19 was reported in São Paulo, Brazil, on February 25, 2020. Other disease hotspots were later identified throughout different LAC countries. Argentina reported the first death on March 7, 2020 (2).

In adults, COVID-19 is characterized by respiratory system involvement, which, in most severe cases, progresses with pneumonia and severe acute respiratory syndrome. For reasons that are not yet fully known, the infection of COVID-19 among children and adolescents is milder, and lethality is much lower (2). Children ages 5 to 14 make up 6.3% of global cases (3).

A particularly vulnerable population is children with cancer, whose mortality from COVID-19 has been reported at 3.6% globally (4).

Studies describing the manifestations of COVID-19 in children are still scarce, and the number of reported cases is often small; however, cases in children with cancer are not reported homogeneously and mostly come from cross-sectional studies (5).

Latin America is a peculiar and heterogeneous region of the planet, where overcrowding in families can be considered a risk factor for the transmission of respiratory diseases, including COVID-19 (6).

According to 2020 reports from the Economic Commission for Latin America and the Caribbean (ECLAC), the pandemic has deepened structural inequalities and poverty. Latin America accounts for only 8.4% of the world population but recorded 27.8% of COVID-19 deaths in 2020 (7).

Moreover, high-impact international journals published fewer reports on the region compared to the United States and European countries (8, 9).

### Objectives

This study aims to determine COVID-19 mortality rates in LAC pediatric cancer patients *via* three routes.

1. Systematic Review.
2. Censuses Published on International Pages.
3. Direct Communication With Willing Pediatric Oncologists From Hospitals That Treat Patients With Cancer and COVID-19 in Mexico, Argentina, Bolivia, and Peru.

## Materials and methods

**Type of study:** Systematic review

Information was retrieved from available indexed articles, registries, or institutional reports on COVID-19 cases in LAC cancer patients 0–18 years old.

Data collection was conducted in two ways:

A review of published literature was performed. The data collection period was from June 21, 2021, to January 7, 2022. PubMed, Google Scholar, and Cochrane Library search engines were used as well as engines such as BIREME, Scielo, and MEDES for literature published in Latin American countries. Keywords and MESH terms (children, pediatrics, oncology, cancer, COVID-19, SARS-CoV-2, complications, mortality, survival) were used.

Published cross-sectional and cohort studies that included cases of COVID-19 in LAC cancer patients were considered. Studies that did not indicate cancer diagnosis and only displayed immunosuppression and those that did not detail the complications or did not clarify the outcome were excluded.

Official worldwide registries (Global COVID-19 registry) were included (4).

The second data collection strategy was retrieving direct census reports from various hospitals in LAC that collaborated with our request to share information.

### Participants

Patients under 18 years of age with a cancer diagnosis and a positive test result for SARS-CoV-2 (by PCR) from LAC were included. Patients who underwent a transplant or patients with cancer surveillance for more than 5 years were excluded.

### Variables

Studies that used the PCR test to diagnose the disease in LAC were included.

The exposure variable was cancer type, grouped as hematological cancer (acute leukemias and lymphomas) and solid tumors (central nervous system tumors, germ cell tumors, hepatoblastoma, neuroblastoma, osteosarcoma, retinoblastoma, sarcomas, rhabdomyosarcoma, and Wilms tumor).

The mortality rate was calculated.

### Outcomes

The outcomes recorded were mortality and critical care admission.

### Searches, strategy, and data extraction

For data collection and extraction, two reviewers manually and independently assessed studies' eligibility for inclusion; no automation tools were used. Relevant studies were retrieved, and the necessary information was subsequently extracted on the characteristics of the included studies about participants, types of complications, and outcome variables. In both phases (study selection and data extraction), the reviewers resolved disagreements by consensus; if disagreement persisted, a third reviewer was consulted.

Information was retrieved from the Global Registry of COVID-19 in Childhood Cancer using the snowball technique.

The data obtained was integrated into evidence tables with the verification of the two reviewers. The collection of the following information was considered: country, totals reported by country, hematological, extracranial, central nervous system tumors, neutropenia, previous lung injury, obesity, chemotherapy for more than 30 days, asymptomatic, mild COVID, moderate COVID, severe COVID, critical COVID, hospitalized, ICU, intubated, intermediate, chemotherapy suspended, reduced, and death and lethality.

#### Information on Latin American Hospitals

The searches were complemented with the information provided by different Latin American hospitals. They were invited to collaborate *via* email and asked to fill out a form with the previously described variables of the cases treated.

The results provided by the LAC collaborating hospitals were integrated into the evidence table, indicating the cases reported by hospitals and those reported in the literature to avoid duplication bias.

### Statistical analysis

Quantitative synthesis was performed with rates calculated using the formula n (number of cases)/total population and expressed in cases per 100 patients with cancer and SARS-CoV-2.

Meanings and conclusions are extracted from unstructured and heterogeneous data organized in evidence tables.

## Results

We collected data from June 21, 2021, to January 7, 2022. Information was retrieved from 14 Latin American countries: Argentina, Bolivia, Brazil, Chile, Colombia, Ecuador, El Salvador, Guatemala, Honduras, Mexico, Panama, Paraguay, Peru, and Uruguay.

There were 1,578 articles retrieved from databases and one registry; of those, 1,510 were eliminated due to duplication, leaving 68 articles. After screening, 23 were excluded, leaving 45 potentially eligible. When reading the complete text, 31 were excluded because they did not contain the full and necessary information for the evidence tables.

Finally, 14 articles were included: two from Argentina, four from Brazil, one from Colombia, five from Mexico, and two from Peru (10–22).

**Figure 1:**
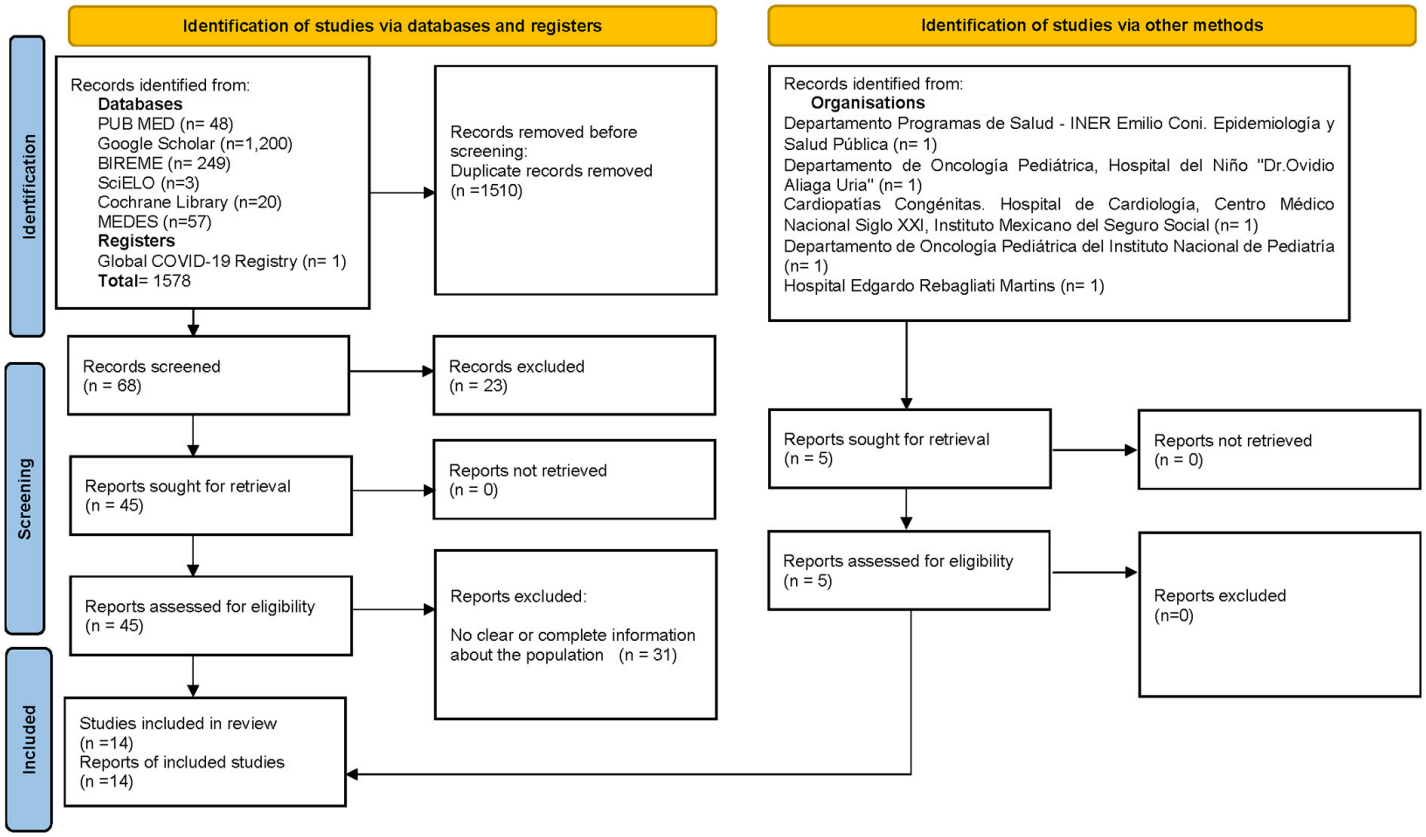
Search flowchart.

Record requests made *via* email to hospitals in the region yielded five records: one from Argentina, one from Bolivia, two from Mexico, and one from Peru. All of the reports complied with the requested information, so they were included (Figure 1).

There were 522 pediatric patients with cancer and diagnosis of COVID-19 (from 14 indexed articles) stored. The mean number of patients per article was 37, with an extensive range of 2–179 for each study. The paper with more patients (15) belongs to a study from Brazil, reporting 12.2% mortality. The articles with fewer patients (16, 18) come from Peru and Mexico and correspond to cross-sectional reports that share experiences from a single center each; also, they do not report deaths (Table 1).

**Table 1:** Patients, mortality and lethality.

**References**	**Country**	**Number of** **patients reported**	**Raw mortality**	**Lethality (%)**
Corso et al. (15)	Brazil	179	22	12.2
Flores et al. (18)	Mexico	2	0	0
Fonseca et al. (19)	Colombia	32	2	6.25
Gentile et al. (23)	Argentina	40	1	2.5
Cleto-Yamane et al. (20)	Brazil	42	0	0
Llaque-Quiroz et al. (16)	Peru	2	0	0
López-Aguilar et al. (14)	Mexico	14	1	7.1
Lima et al. (24)	Brazil	48	8	16.6
Montoya Jaqueline et al. (12)	Peru	69	7	10.1
Olivar-López et al. (11)	Mexico	17	0	0
Palomo-Collí et al. (22)	Mexico	38	0	0
Prata-Barbosa et al. (17)	Brazil	4	0	0
Sánchez-Jara et al. (10)	Mexico	15	7	46.6
Schönfeld et al. (13)	Argentina	20	3	15
Total		522	51	9.8

As a result, the heterogeneity in mortality attributed to COVID-19 stands out. While global mortality is reported at 3.6% (4), the table shows that the mortality percentage per published study varies widely, from 0 to 46.6% in the study that reported the most deaths attributed to COVID-19 (10).

It is noteworthy that six of the 14 analyzed studies (42.8% of the total articles) report no mortality; however, the mean mortality of 522 patients in this work gives an average of 9.8%, well above the number mentioned in the global analyses.

For the analysis of all the indexed studies, 522 patients were included, with a Fatality Rate (FR) of 9.8 per 100 patients with SARS-CoV-2 and cancer. Brazil reported the most significant number of subjects (*n* = 273) in four studies, with a FR of 11 per 100 patients.

The country analysis of the Global Registry of COVID-19 in Childhood Cancer included information from 13 LAC (except Bolivia) with 744 registered patients and an FR of 3.2 per 100 patients. The country with the highest FR was Brazil, with 11 per 100 patients.

The gray literature (clinical records analysis of participating institutions from four countries), 296 subjects were included, with an FR of 8.8 per 100 patients, and Mexico (*n* = 131) accumulated an FR of 13.7 per 100 patients (Table 2).

**Table 2:** Analysis including indexed studies, clinical records and others (gray literature) of mortality and SARS-CoV-2 in pediatric patients and cancer in Latin America.

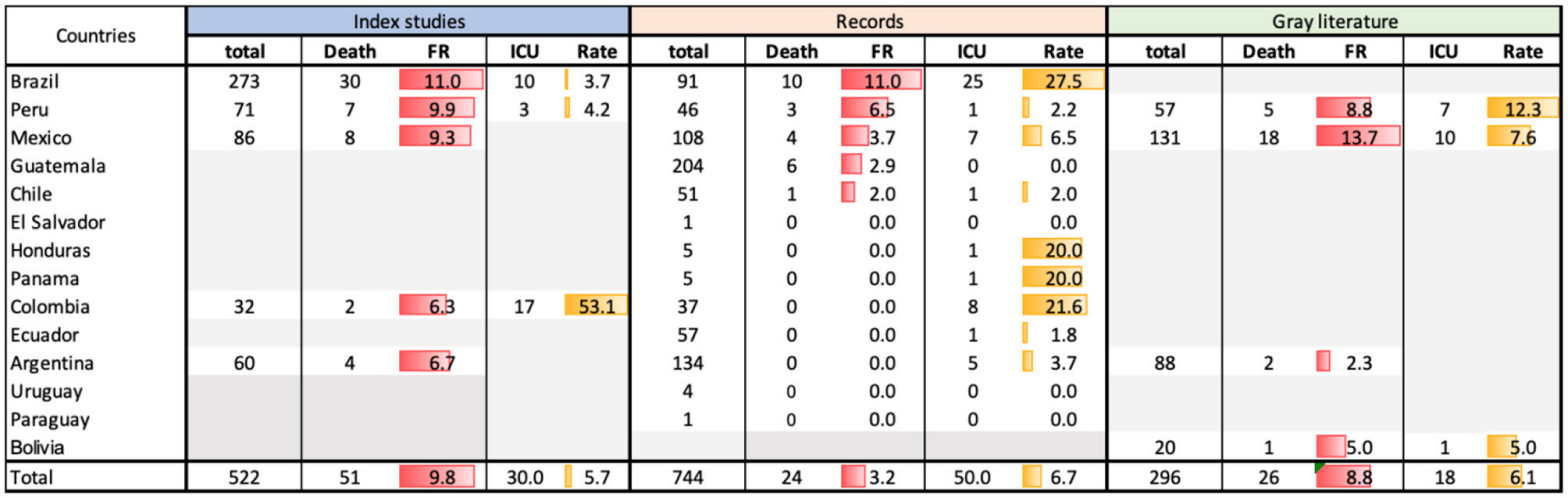

Records: Global Registry of COVID-19 in Childhood Cancer. St Jude; Gray literature: include clinical records from participating countries.

FR, Fatality rate, ICU, intensive care unit. Red color indicates fatality rate and yellow color indicates ICU rate.

The rates of patients in the ICU were 5.7 in the indexed records, 6.7 in the international electronic reports, and 6.1 in the hospital censuses collected.

**Figure 2:**
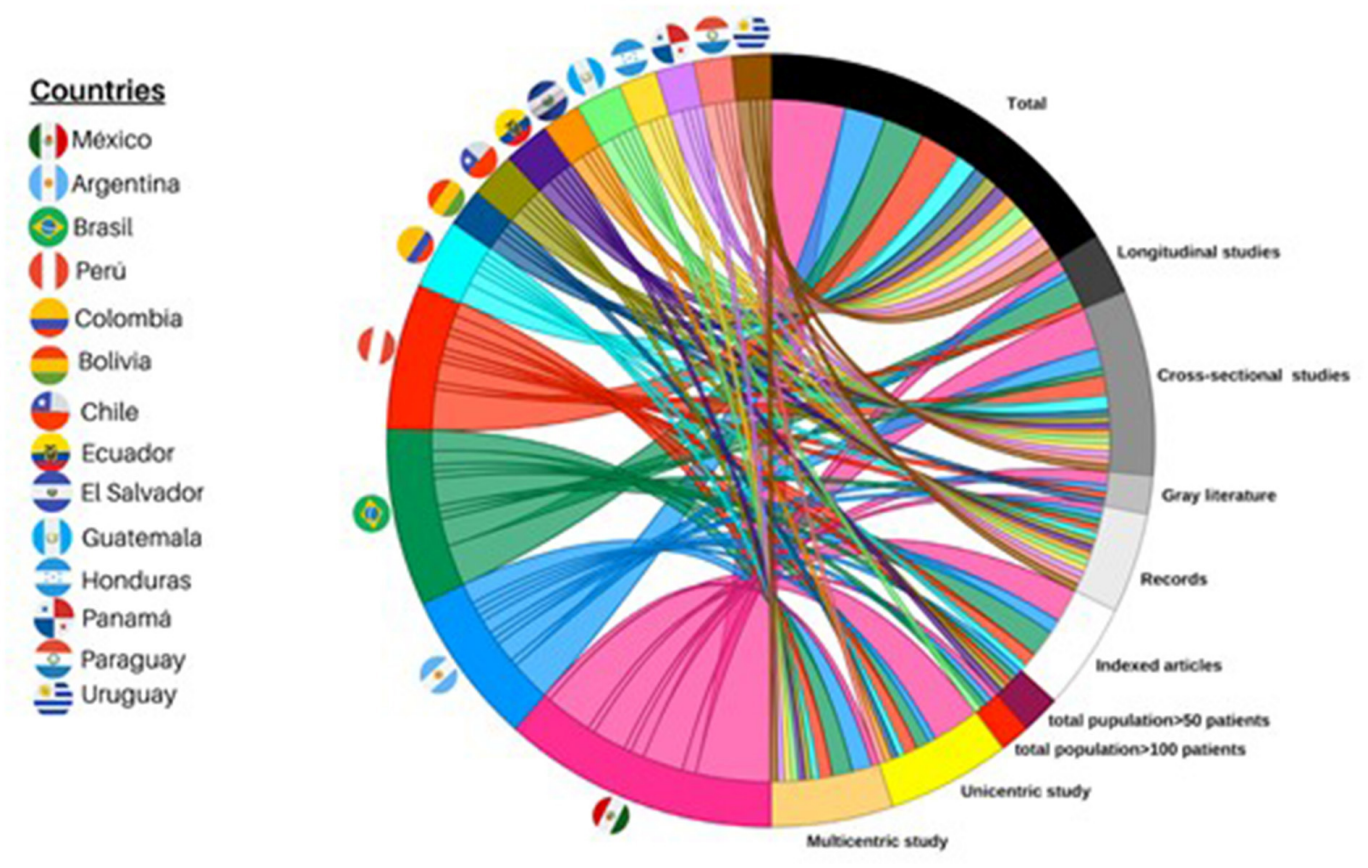
Chordal graph, information characteristics. A chord diagram is constructed from the information. Half circle shows each country with a specific color (for example, Mexico is pink); Each string is directed to a tape representing a variable (for example, longitudinal studies). The thicker the string, the greater the number of studies it means.

Mexico and Brazil had the highest production of published data. Brazil is the country with the most significant number of longitudinal studies. And the countries with the most multicenter studies were Brazil and Argentina (Figure 2).

## Discussion

Priority has been given to spreading information related to COVID-19 globally. In search engines, developed countries stand out with a more significant number of publications associated with COVID-19 and its impact on pediatric cancer patients than countries of the LAC.

At the beginning of the pandemic, the reports on this topic globally were heterogeneous, with a lack of follow-up, or were simply preliminary reports or letters to the editor, so it wasn't possible to carry out a meta-analysis. Over time, the quality of the literature improved, and it was possible to get better-quality articles with data from developed countries (25).

On the other hand, the currently reported information makes it impossible to have methodological rigor in the LAC, making it not feasible to conduct a meta-analysis.

This review included papers in which PCR confirmed the diagnosis of COVID-19 as the gold standard.

To avoid selection bias, articles that mentioned the type of neoplasm, and not only neoplasm with immunosuppression, were included.

The information was analyzed separately to avoid repeated information bias between the articles published in the institutes' databases.

The reporting bias could have more weight in this review since reports from institutions in a certain period are included. Unfortunately, the records of at least one institution per Latin American country were not available.

Reporting biases, the high fatality rates according to hospital censuses, and the differences in the quantity and quality of reports originating in LAC all seem typical for the region.

The rate of critically ill patients who have received intensive care is very similar in indexed and non-indexed registries (5.7 and 6.7%, respectively). However, these numbers contrast with those reported in the literature for pediatric patients without cancer. The literature states that the rate of such patients needing the ICU is 2.9% (26).

Due to the pandemic, a more significant investment in resources was required to reorganize the health system to respond to the crisis. In many cases, there was a displacement of resources from primary healthcare services to be able to attend to the COVID-19 disease (27).

The main affectation in terms of child health in this region is not caused by the risk of contracting COVID-19 but by the suspension and delay in access to health, which affects timely diagnosis, treatment, and rehabilitation; in addition to delaying preventive actions and early detection of other preventable or treatable diseases (28).

Latin America did not present a particular medical care strategy but hospital reconversion programs (29).

This data should be considered for hospital resource planning in places that care for children with cancer.

According to ECLAC, these areas will face significant pandemic-related challenges in 2022. Such challenges will affect social costs, economic growth, and job creation, and this year the region will experience only a third of the growth that was anticipated in 2021. This area of the world will be hit the hardest by the pandemic (30). The region's economy grew by 6.2% in 2021, but it will expand only 2.1% this year, ECLAC added. According to Alicia Bárcena (ECLAC Executive Secretary), “Today we are in the process of clear divergence and degrees of asymmetry. The advanced economies would be the only ones to resume the [anticipated] growth path in 2022. The emerging economies would only resume these trends in 2025 (31).”

The chances of suffering from a severe or critical illness due to COVID-19 were almost six times higher in Latin America than in high-income countries.

Public spending on health in the region is 3.8%, below the 6.6% that goes to health in OECD countries.

Only Cuba and Uruguay are above 6%, and countries such as Argentina, Costa Rica, and Colombia are close. The rest of the Latin American countries are far from the expected public spending on health.

In Latin America, government coverage in health and insurance is also lower (54.3%) compared to the OECD (73.6%).

The redistribution of public spending, as well as more efficient use and avoiding waste, can improve health security, the effectiveness of care, and the quality of life of the population in this region (32).

The year 2022 will bring significant challenges in terms of economic growth, job creation, and the social toll of the pandemic.

The collected information and various ways in which the results were reported have made it impossible to perform a meta-analysis. Most of the studies are susceptible to selective reporting bias because the hospital centers responsible for the publications overwhelmingly treated cancer patients. There is a risk of follow-up bias since most were cross-sectional studies, and a causal association cannot be made. In addition, associated exposure variables—such as the cancer type, therapeutic phase, coexistence with a fever event, and neutropenia—were not reported in most publications.

Other data sources, such as national registries, inconveniently note that cancer was not recorded as a comorbidity but rather was grouped with other diseases that cause immunosuppression (33).

The strength of this work lies in exposing the disparity in the scientific reports of LAC countries compared to those of other continents.

## Conclusions

This systematic review is the first effort to document the situation of children with cancer and COVID-19 in the LAC region. The contrast between countries in the area is manifested when reporting the cases and preparing scientific articles. Furthermore, when this data is compared with the records and papers published in developed countries, the quality of the information seems poor.

Due to the aforementioned, the information reported should be taken with reservation since all of the biases of observational studies are present.

In this sense, the countries of this region should standardize and improve their documentation of these cases to gain greater knowledge about the situation. The information generated will thus be helpful for doctors and decision-makers.

Data taken from Global Health Intelligence, shows that 15% of hospitals have a Tele Medicine (TM) program; 9% are connected to a TM center, and just 6% of them provide that service to patients through a medical monitoring system and 1 percent are part of international TM. These data remained stable between 2020 and 2021. TM is an opportunity area in Latin America (34).

Another critical point is the lack of massive vaccinations among pediatric patients in the region, for which collective action is required.

Vaccination looks promising in this group of children. Due to the extreme vulnerability of cancer patients to COVID-19, children above 12 years old can be vaccinated 3 to 7 days after chemotherapy.

Vaccinations are expected to reduce the risk of therapy interruptions and the incidence of COVID-19 complications. The long-term prognosis of treated patients is unclear due to the short observation period. The effects on pediatric cancer curability must be analyzed in the near and long-term future, but according to observations in some cooperative groups interruptions in therapy are common, and current data indicate that the issue of suboptimal treatment in COVID-19 survivors can be addressed in the future (35).

It is also essential to mention that the search for information was carried out before vaccinations were implemented in many LAC countries; this variable will indeed play an important role in subsequent research.

## Data availability statement

The original contributions presented in the study are included in the article/supplementary material, further inquiries can be directed to the corresponding author/s.

## Author contributions

ED-A, DÁ-M, and HM-G: manuscript writing. JD, PP-N, LV-H, SA, LG-R, and LJ-V: substantial contributions and manuscript review. All authors contributed to the article and approved the submitted version.

## Conflict of interest

The authors declare that the research was conducted in the absence of any commercial or financial relationships that could be construed as a potential conflict of interest.

## Publisher's note

All claims expressed in this article are solely those of the authors and do not necessarily represent those of their affiliated organizations, or those of the publisher, the editors and the reviewers. Any product that may be evaluated in this article, or claim that may be made by its manufacturer, is not guaranteed or endorsed by the publisher.
